# Polyhexamethylene Guanidine Phosphate Induces Restrictive Ventilation Defect and Alters Lung Resistance and Compliance in Mice

**DOI:** 10.3390/toxics12110776

**Published:** 2024-10-25

**Authors:** Yoon Hee Park, Sang-Hoon Jeong, Hong Lee, Yoon-Jeong Nam, Hyejin Lee, Yu-Seon Lee, Jin-Young Choi, Su-A Park, Mi-Jin Choi, Hayan Park, Jaeyoung Kim, Eun-Yeob Kim, Yong-Wook Baek, Jungyun Lim, Sua Kim, Je-Hyeong Kim, Ju-Han Lee

**Affiliations:** 1 Medical Science Research Center, Korea University Ansan Hospital, Korea University College of Medicine, 123 Jeokgeum-ro, Danwon-gu, Ansan-si 15355, Gyeonggi-do, Republic of Korea; 2 Environmental Health Research Division, National Institute of Environmental Research, Incheon 22689, Gyeonggi-do, Republic of Korea; 3 Department of Critical Care Medicine, Korea University Ansan Hospital, Korea University College of Medicine, 123 Jeokgeum-ro, Danwon-gu, Ansan-si 15355, Gyeonggi-do, Republic of Korea; 4 Division of Pulmonary and Critical Care Medicine, Department of Internal Medicine, Korea University Ansan Hospital, Korea University College of Medicine, 123 Jeokgeum-ro, Danwon-gu, Ansan-si 15355, Gyeonggi-do, Republic of Korea; 5 Department of Pathology, Korea University Ansan Hospital, Korea University College of Medicine, 123 Jeokgeum-ro, Danwon-gu, Ansan-si 15355, Gyeonggi-do, Republic of Korea

**Keywords:** polyhexamethylene guanidine phosphate, pulmonary function test, fibrosis, mouse

## Abstract

Polyhexamethylene guanidine phosphate (PHMG-p), a major ingredient of humidifier disinfectants, is known to induce inflammation, interstitial pneumonitis, and fibrosis in the lungs. While its histopathologic toxicities have been studied in rodents, research on pulmonary function test (PFT) changes following PHMG-p exposure is limited. This study aimed to investigate the acute and chronic effects, as well as the dose and time response, of PHMG-p on the lungs in mice using PFT and histopathologic examinations. In the single instillation model, mice received PHMG-p and were sacrificed at 2, 4, and 8 weeks. In the five-time instillation model, PHMG-p was administered five times at one-week intervals, and mice were sacrificed 10 weeks after the first instillation. Results showed that PHMG-p exposure reduced lung volume, increased resistance, and decreased compliance, indicating a restrictive ventilation defect. Histopathologic examination showed increases in lung inflammation and fibrosis scores. Changes in several lung volume and compliance parameters, as well as histopathology, were dose-dependent. Lung resistance and compliance parameters had significant correlations with lung inflammation and fibrosis scores. PHMG-p exposure in mice resulted in a restrictive ventilation defect with altered lung resistance and compliance, along with histopathologic lung inflammation and fibrosis.

## 1. Introduction

Humidifier disinfectants (HDs) were widely used in South Korea from 2006 to 2011 to inhibit microorganism growth in humidifier water tanks. During this period, several cases of unexplained fatal lung diseases began to be reported among HD users [1]. Nationwide surveys subsequently identified humidifier disinfectant-associated lung injury (HDALI), characterized by interstitial pneumonitis and widespread lung fibrosis [2]. Approximately 4179 cases have been officially recognized as HDALI, with an estimated 11 million potential victims [3]. Polyhexamethylene guanidine phosphate (PHMG-p), a major ingredient in HDs, effectively prevented bacterial and fungal growth [4,5]. However, PHMG-p has been reported to induce pulmonary toxicities, including lung inflammation and fibrosis, in rodent models [6,7,8,9,10]. Furthermore, PHMG-p has been shown to cause genetic changes and trigger the expression of toxicity-related factors in cell lines [11,12,13]. Prolonged exposure to PHMG-p has also been linked to lung cancer in rats and genetic alterations associated with lung cancer in pulmonary epithelial cells [5,14,15].

The pulmonary function test (PFT) has been essential for evaluating and managing respiratory health and diseases, especially in determining the severity of pulmonary fibrosis [16,17,18], such as HDALI. Despite the challenges posed by the need for cooperation in animal experiments, a system for conducting PFT in rodents has been developed and is now in use [19]. Rodent PFT systems are highly sensitive tools for studying lung function in response to toxic substances and materials that cause lung diseases in mouse models, thus enhancing lung disease research with experimental animals. Although reduced lung functions in PFT have been reported in patients exposed to HDs [20,21], there are very few corresponding studies in animal models.

The objective of this study was to investigate the acute and chronic effects, as well as the dose and time response of PHMG-p on the lungs in mice using PFT and histopathologic examinations.

## 2. Materials and Methods

### 2.1. Experimental Designs

Eight-week-old male C57BL/6 mice (KOATECH, Gyeonggi-do, Republic of Korea) were acclimated for one week and maintained under standard conditions: a temperature of 22–25 °C, relative humidity of 40–60%, and a 12-h light–dark cycle. The mice were provided with pelleted food (Purina, Gyeonggi-do, Republic of Korea) and filtered tap water. All procedures followed the National Institutes of Health Guide for the Care and Use of Laboratory Animals and were carried out at Korea University. The experimental protocols received approval from the Institutional Animal Care and Use Committee of Korea University Medical Center (KOREA-2023-0055-C1).

**Figure 1:**
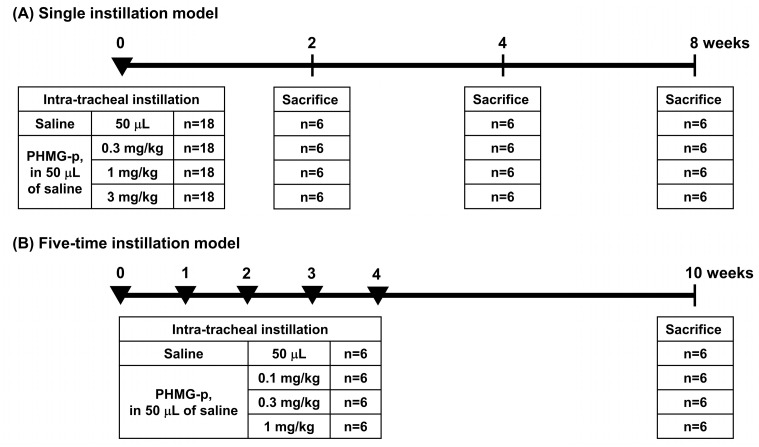
The overall scheme of the experiments. (**A**) Single instillation model. (**B**) Five-time instillation model. PHMG-p: polyhexamethylene guanidine phosphate.

This study utilized two experimental models (Figure 1): single and five-time intratracheal (IT) instillation models. In the single instillation model, mice received either saline (50 μL, JW Pharmaceutical Co., Seoul, Republic of Korea; *n* = 18) or PHMG-p (0.3, 1, or 3 mg/kg in 50 μL of saline; BOC Sciences, Shirley, NY, USA; CAS registry number 89697-78-9; *n* = 18 in each dose subgroup). Six mice each from the saline group and the three PHMG-p dose subgroups were sacrificed at 2, 4, and 8 weeks after instillation (Figure 1A). In the five-time instillation model, saline (50 μL; *n* = 6) or PHMG-p (0.1, 0.3, or 1 mg/kg in 50 μL of saline; *n* = 6 in each dose subgroup) was administered five times at one-week intervals, and mice were sacrificed 10 weeks after the first instillation (Figure 1B). The doses of PHMG-p in the single instillation model were based on a previous study [22]. For the five-time instillation model, doses were established through preliminary experiments, with lower doses set than the single instillation model to mitigate the risk of death due to toxicity from repeated exposure. Mice were anesthetized using isoflurane (2% isoflurane in 70% N_2_O and 30% O_2_). The intubation-mediated intratracheal administration method was employed, where the solution is instilled directly into the lungs via the trachea. The instillation procedures were verified using a modified videoscope developed in-house [23].

### 2.2. PFT

PFT in mice was conducted using a PFT system (DSI, Buxco^®^, St. Paul, MN, USA) and software (DSI, Buxco^®^, FinePointeTM, St. Paul, MN, USA). The system was set up and calibrated according to the DSI PFT manual for mice. Mice were anesthetized with ketamine (50 mg/kg; Yuhan Corporation, Seoul, Republic of Korea) and medetomidine (0.33 mg/kg; Tomidin^®^, Provet, Istanbul, Turkey) via intraperitoneal injection. The tracheal cannula provided by the manufacturer of the PFT system was inserted into the trachea of the anesthetized mice and connected to the PFT system. Using the system and software, a total of 51 parameters were measured through four tests, including functional residual capacity (FRC), quasistatic pressure volume, fast flow volume, and resistance and compliance tests. Among the measurements, the parameters related to lung volume, resistance, and compliance were analyzed.

For lung volume, the measurements included forced expiratory volume in 100 milliseconds (FEV100), forced vital capacity (FVC), FRC, tidal volume (TV), vital capacity (VC), and inspiratory capacity (IC). FEV100/FVC and total lung capacity (TLC) were calculated from the measured lung volume parameters. For resistance and compliance, the measurements included chord compliance (Cchord), compliance at 50% vital capacity (Cfvc50), dynamic compliance (Cdyn), lung resistance (RI), and the maximum pressure change over the breath (dPmax).

### 2.3. Histopathologic Inflammation and Fibrosis Scores

The lungs were perfused with normal saline through the interventricular septum. The mice’s lungs were immediately instilled with 10% neutral buffered formalin through the trachea at a hydrostatic pressure of 15 cm H_2_O. Paraffin sections were cut into 4 μm slices from the fixed samples and then stained with hematoxylin and eosin (H&E) and Masson’s trichrome (MT). Inflammation scores were calculated based on the extent and severity of inflammation. Extent was scored as follows: 0 = none, 1 = lesions involving 0% to 25% of the lung, 2 = lesions involving > 25% to 50% of the lung, and 3 = lesions involving > 50% of the lung. Severity was scored as follows: 0 = none, 1 = mild, 2 = moderate, and 3 = severe. The total inflammation score was obtained by adding the extent and severity scores. A modified Ashcroft score was used to quantify fibrosis [24].

### 2.4. Statistics

All statistical analyses were performed using IBM SPSS statistical software, version 25.0 (IBM Corporation, Armonk, NY, USA). All data are expressed as the mean ± SD. For PFT parameters and histopathologic scores, a one-way analysis of variance (ANOVA) with post hoc Tukey’s Honestly Significant Difference (HSD) test was conducted. The correlation between PFT parameters and histopathologic inflammation and fibrosis scores at 2 weeks in the single instillation model and at 10 weeks in the five-time instillation model was analyzed using Pearson correlation. Statistical differences were considered significant at *p* < 0.05.

## 3. Results

### 3.1. PHMG-p Induced Restrictive Ventilation Defect and Altered Lung Compliance and Resistance in Mice

**Figure 2:**
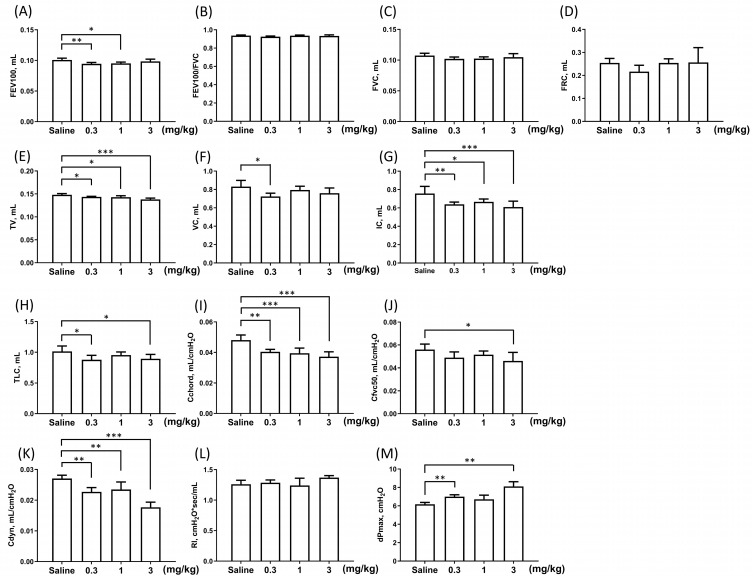
Pulmonary function test results at 2 weeks in the single instillation model. (**A**) Forced expiratory volume in 100 milliseconds, FEV100. (**B**) FEV100/forced vital capacity, FEV100/FVC. (**C**) Forced vital capacity, FVC. (**D**) Functional residual capacity, FRC. (**E**) Tidal volume, TV. (**F**) Vital capacity, VC. (**G**) Inspiratory capacity, IC. (**H**) Total lung capacity, TLC. (**I**) Chord compliance, Cchord. (**J**) Compliance at 50% vital capacity, Cfvc50. (**K**) Dynamic compliance, Cdyn. (**L**) Lung resistance, RI. (**M**) Maximum pressure change over the breath, dPmax. Data are presented as mean ± SD. * *p* < 0.05, ** *p* < 0.01 and *** *p* < 0.001.

In the single instillation model (Table 1), two weeks after administration, FEV100 significantly decreased in the 0.3 and 1 mg/kg PHMG-p subgroups (*p* < 0.05) compared to the saline group (Figure 2A). FEV100/FVC did not differ from the saline group (*p* > 0.05 in all PHMG-p subgroups; Figure 2B). FVC showed a tendency to decrease in all PHMG-p subgroups, though this change was not significant (*p* > 0.05 in all PHMG-p subgroups; Figure 2C). TV (*p* < 0.05 in all PHMG-p subgroups; Figure 2E), VC (*p* = 0.009 in the 0.3 mg/kg PHMG-p subgroup; Figure 2F), IC (*p* < 0.05 in all PHMG-p subgroups; Figure 2G), and TLC (*p* < 0.05 in the 0.3 and 3 mg/kg PHMG-p subgroups; Figure 2H) were significantly decreased, with the reductions in TV being dose-dependent. These decreases in lung volume parameters, along with a maintained FEV100/FVC, indicated a restrictive ventilation defect due to PHMG-p instillation. Among the resistance and compliance parameters, Cchord (*p* < 0.01 in all PHMG-p subgroups; Figure 2I), Cfvc50 (*p* = 0.020 in the 3 mg/kg PHMG-p subgroup; Figure 2J), and Cdyn (*p* < 0.01 in all PHMG-p subgroups; Figure 2K) significantly decreased compared to the saline group, with dose-dependent changes in Cchord and Cdyn. RI showed a slight increase in the 3 mg/kg PHMG-p subgroup (*p* = 0.078; Figure 2L). However, dPmax (*p* < 0.01 in the 0.3 and 3 mg/kg PHMG-p subgroups; Figure 2M) significantly increased at 2 weeks. The decreases in Cchord, Cfvc50, and Cdyn, along with the increases in RI and dPmax, reflected decreased lung compliance and increased.

Lung resistance. By 4 and 8 weeks, differences in FEV100, TV, VC, IC, TLC, Cchord, Cfvc50, Cdyn, RI, and dPmax among the PHMG-p instillation subgroups were no longer significant (Table 1).

**Figure 3:**
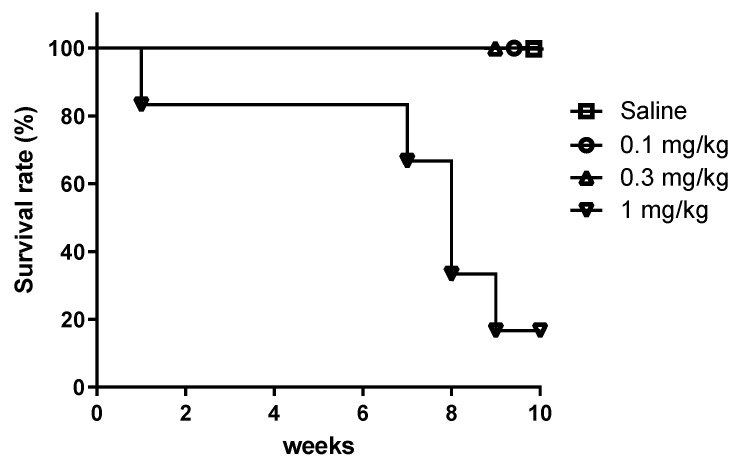
Survival rate of mice for PHMG-p instillation. In the groups administered saline, 0.1, and 0.3 mg/kg of PHMG-p, all mice survived for up to 10 weeks, but in the group administered a high concentration of 1 mg/kg of PHMG-p, only one out of six survived at 10 weeks.

**Figure 4:**
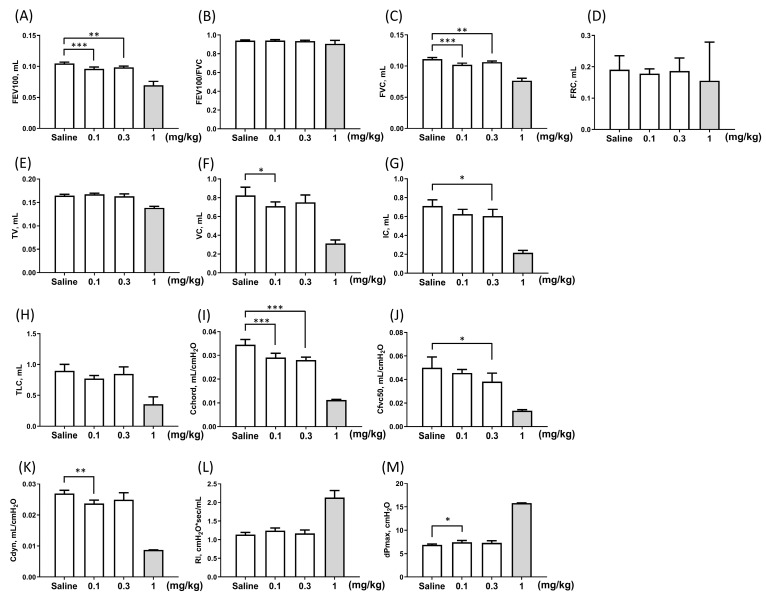
Pulmonary function test results at 10 weeks in the five-time instillation model. (**A**) Forced expiratory volume in 100 milliseconds, FEV100. (**B**) FEV100/forced vital capacity, FEV100/FVC. (**C**) Forced vital capacity, FVC. (**D**) Functional residual capacity, FRC. (**E**) Tidal volume, TV. (**F**) Vital capacity, VC. (**G**) Inspiratory capacity, IC. (**H**) Total lung capacity, TLC. (**I**) Chord compliance, Cchord. (**J**) Compliance at 50% vital capacity, Cfvc50. (**K**) Dynamic compliance, Cdyn. (**L**) Lung resistance, RI. (**M**) Maximum pressure change over the breath, dPmax. Data are presented as mean ± SD. * *p* < 0.05, ** *p* < 0.01 and *** *p* < 0.001.

In the five-time instillation model (Table 1), particularly in the 1 mg/kg PHMG-p subgroup, five out of six mice died within 10 weeks following instillation, leaving only one mouse surviving. PFTs were conducted twice on the surviving mouse, and the averaged results were analyzed. The survival curve for mice is shown in Figure 3. Although data from the 1 mg/kg PHMG-p subgroup at 10 weeks were presented (Figure 4), the data were excluded from the statistical analysis to avoid potential bias. At 10 weeks, significant impairments were observed in FEV100 (*p* < 0.01 in the 0.1 and 0.3 mg/kg PHMG-p subgroups; Figure 4A), and FVC (*p* < 0.01 in the 0.1 and 0.3 mg/kg PHMG-p subgroups; Figure 4C) compared with the saline group, while FEV100/FVC (*p* > 0.05 in the 0.1 and 0.3 mg/kg PHMG-p subgroups; Figure 4B) was not different with the saline group. VC (*p* = 0.042 in the 0.1 mg/kg PHMG-p subgroup; Figure 4F) and IC (*p* = 0.025 in the 0.3 mg/kg PHMG-p subgroup; Figure 4G) were significantly decreased. These results also indicated the development of a restrictive ventilation defect caused by PHMG-p instillation. In the resistance and compliance parameters, Cchord (*p* < 0.001 in the 0.1 and 0.3 mg/kg PHMG-p subgroups; Figure 4I), Cfvc50 (*p* = 0.024 in the 0.3 mg/kg PHMG-p subgroup; Figure 4J), and Cdyn (*p* = 0.007 in the 0.1 mg/kg PHMG-p subgroup; Figure 4K) significantly decreased compared with the saline group, with dose-dependent changes in Cchord. dPmax (*p* = 0.039 in the 0.1 mg/kg PHMG-p subgroup; Figure 4M) significantly increased. These changes in Cchord, Cfvc50, and Cdyn, along with the increase in dPmax, indicated decreased lung compliance and increased lung resistance.

### 3.2. PHMG-p Caused Dose-Dependent Lung Inflammation and Fibrosis in Mice

**Figure 5:**
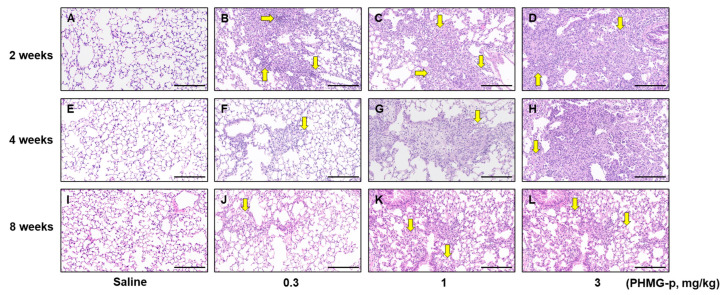
Histopathologic changes at 2, 4, and 8 weeks in the single instillation model (H&E, ×200). The bar at the bottom of each figure indicates 200 μm. Saline (**A**), 0.3 mg/kg (**B**), 1 mg/kg (**C**), and 3 mg/kg (**D**) at 2 weeks. Saline (**E**), 0.3 mg/kg (**F**), 1 mg/kg (**G**), and 3 mg/kg (**H**) at 4 weeks. Saline (**I**), 0.3 mg/kg (**J**), 1 mg/kg (**K**), and 3 mg/kg (**L**) at 8 weeks. Saline groups showed normal alveoli and well-preserved lung architecture (**A**,**E**,**I**). Mixed inflammatory cells (arrows) and fibrosis were seen in PHMG-treated groups (**B**–**D**,**F**–**H**) at 2 and 4 weeks. Foamy histiocytes (arrows) and mild fibrosis were seen in PHMG-treated groups (**J**–**L**) at 8 weeks.

**Figure 6:**
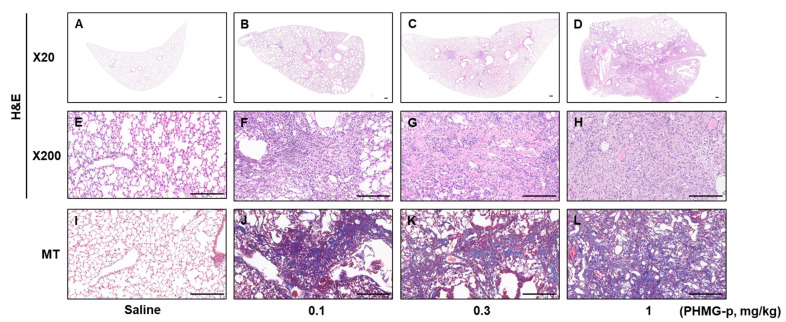
Histopathologic changes at 10 weeks in the five-time instillation model. The bar at the bottom of each figure indicates 200 μm. Low power images (H&E, ×20) of saline (**A**), 0.1 mg/kg (**B**), 0.3 mg/kg (**C**), and 1 mg/kg (**D**). H&E images (×200) of saline (**E**), 0.1 mg/kg (**F**), 0.3 mg/kg (**G**), and 1 mg/kg (**H**). Masson Trichrome (MT) images (×200) of saline (**I**), 0.1 mg/kg (**J**), 0.3 mg/kg (**K**), and 1 mg/kg (**L**). Saline groups showed normal alveoli and well-preserved lung architecture (**A**,**E**,**I**). The extent and severity of fibrosis of PHMG were dose-dependent (**B**–**D**,**F**–**H**,**J**–**L**).

**Figure 7:**
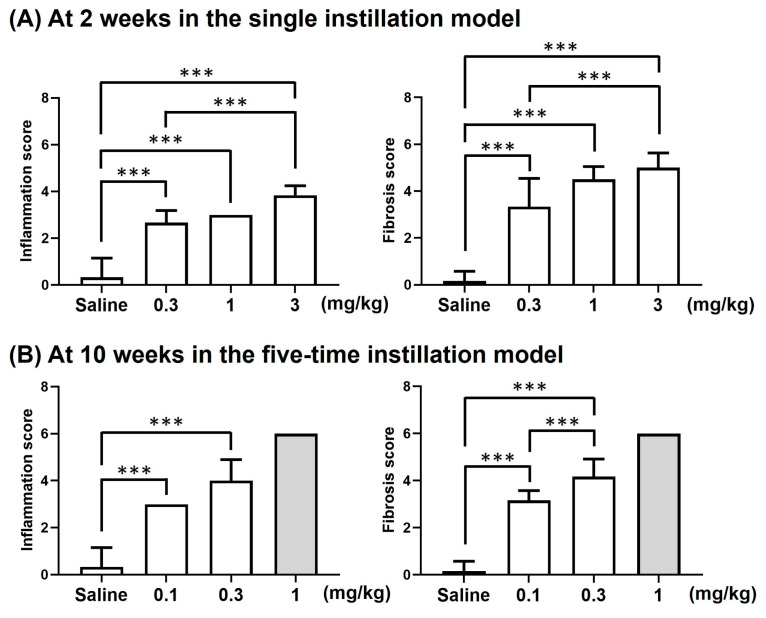
Inflammation and fibrosis scores at 2 weeks in the single instillation model and at 10 weeks in the five-time instillation model. Polyhexamethylene guanidine phosphate induced lung inflammation and fibrosis dose-dependently in both models. Data are presented as mean ± SD. *** *p* < 0.001.

Saline treatment in mice did not induce any abnormalities in lung tissues at 2, 4, and 8 weeks in the single instillation model, nor at 10 weeks in the five-time instillation model (Figure 5 and Figure 6). In the single instillation model, PHMG-p caused significant inflammation and fibrosis at 2 (*p* < 0.001 in all PHMG-p subgroups), 4 (*p* < 0.05 in all PHMG-p subgroups), and 8 weeks (*p* < 0.05 in all PHMG-p subgroups) (Figure 5 and Figure 7A, Table 2). The inflammation and fibrosis were dose-dependent and most severe at 2 weeks, with a slight tendency for recovery at 4 and 8 weeks (Figure 5 and Figure 7A, Table 2). At 10 weeks in the five-time instillation model, the fibrosis and inflammation were dose-dependently extensive (*p* < 0.001 in 0.1 and 0.3 mg/kg PHMG-p subgroups) compared to the saline group (Figure 6 and Figure 7B, Table 3).

### 3.3. Correlation Between Resistance and Compliance Parameters and Histopathologic Scores

Correlation analyses between resistance and compliance parameters and histopathologic scores were conducted for measurements at 2 weeks in the single instillation model and at 10 weeks in the five-time instillation model (Table 4). In the single instillation model, Cchord, Cfvc50, and Cdyn exhibited significant negative correlations with both inflammation and fibrosis scores (*p* < 0.01 for all comparisons). dPmax demonstrated a significant positive correlation with inflammation (r = 0.659, *p* < 0.001) and fibrosis (r = 0.737, *p* < 0.001) scores, whereas RI showed no correlation with inflammation (r = 0.212, *p* = 0.321) or fibrosis (r = 0.348, *p* = 0.095) scores. In the five-time instillation model, Cchord, Cfvc50, and Cdyn also showed significant negative correlations with inflammation and fibrosis scores (*p* < 0.01 for all comparisons), and RI and dPmax displayed positive correlations (*p* < 0.01 for all comparisons).

## 4. Discussion

In this study, IT instillation of PHMG-p, a major ingredient of HDs, induced a significant restrictive ventilation defect, decreased lung compliance, and increased lung resistance in PFT parameters, along with histopathologic lung inflammation and fibrosis. These changes were evident at 2 weeks in the single instillation model and at 10 weeks in the five-time instillation model. However, at 4 and 8 weeks in the single instillation model, the changes in PFT parameters had recovered despite the presence of histopathologic lung inflammation and fibrosis. Changes in several lung volume and compliance parameters, as well as histopathology, were dose-dependent. Lung resistance and compliance parameters had significant correlations with lung inflammation and fibrosis scores.

Common clinical symptoms among victims of HDALI include shortness of breath and hypoxemia [25,26]. HD-induced interstitial pneumonitis and lung fibrosis have been reported as the underlying histopathological changes responsible for these symptoms [27]. PFT among victims of HDALI is characterized by a restrictive ventilation defect with a reduction in lung volumes and diffusion abnormalities [20,21]. The histopathological changes have been investigated in many animal studies on HDALI [14,28]. However, studies on PFT in animals are very limited, and even in those that exist, only a few parameters have been examined with crude methods [29]. Therefore, this study aimed to investigate the overall changes in PFT, including lung volumes, resistance, and compliance parameters, using an advanced rodent PFT system.

The changes in lung volume parameters have been examined in other animal studies. FEV100, which is analogous to forced expiratory volume in the first second used in human PFT [30], forced expiratory volume in 200 milliseconds (FEV200) [31], and TV [32] have been reported to be reduced in models of lung inflammation and fibrosis. In this study, FEV100 decreased significantly due to PHMG-p at 2 weeks in the single instillation model and at 10 weeks in the five-time instillation model. FVC showed a decreasing trend in the single instillation model and a significant reduction in the five-time instillation model, resulting in a maintained FEV100/FVC ratio similar to other studies on pulmonary fibrosis [31,33]. TV (not in the five-time instillation model), VC, IC, and TLC (not in the five-time instillation model) were also significantly decreased. These general changes in lung volume parameters clearly demonstrated that PHMG-p causes a restrictive ventilation defect.

The resistance and compliance are altered with the development of lung inflammation and fibrosis. Among the PFT parameters of compliance, Cchord (a measure to assess the elasticity or flexibility of the lung tissues), Cfvc50 (a parameter to measure lung compliance at a specific point during forced exhalation), and Cdyn have been reported to decrease in lung inflammation and fibrosis [16,34,35]. This study also showed that PHMG-p reduced Cchord, Cfvc50, and Cdyn at 2 weeks in the single instillation model and at 10 weeks in the five-time instillation model. As the parameters reflect resistance, dPmax and RI were measured. dPmax increased significantly, but RI showed only an increasing trend or remained unchanged. These results indicated decreased lung compliance and increased lung resistance caused by PHMG-p.

This study utilized both single/acute (the single instillation) and repeated/chronic (the five-time instillation) exposure models. In the single/acute exposure model, PHMG-p was instilled once, and PFT and histopathologic changes were measured and observed at 2, 4, and 8 weeks. In the repeated/chronic exposure model, PHMG-p was instilled five times, and chronic changes in PFT and histopathology were measured and observed at 10 weeks. The changes in PFT and histopathology in the repeated/chronic exposure model were consistent with the expected findings reported among victims of HDALI [20,21], which develops from chronic exposure to HDs. However, in the single/acute exposure model, the initial significant alterations in PFT parameters resolved over time, and the degree of histopathologic changes showed tendencies of recovery. These results were unexpected, and although the exact mechanism cannot be explained, they may provide a partial explanation for persons who were temporarily exposed to HDs but did not progress to clinical HDALI.

To evaluate the dose–response effect of PHMG-p on PFT and histopathology, various doses of PHMG-p were used in both the single/acute and repeated/chronic exposure models. In the single/acute exposure model, doses of 0.3, 1, or 3 mg/kg of PHMG-p were instilled, and doses of 0.1, 0.3, or 1 mg/kg of PHMG-p were instilled in the repeated/chronic exposure model. The doses for the repeated/chronic exposure model were set lower than those for the single/acute exposure model to mitigate the risk of death due to toxicity from repeated exposure. In the PFT, dose-dependent decreases were observed in TV, Cchord, and Cdyn at 2 weeks in the single instillation model and in Cchord at 10 weeks in the five-time instillation model. Although the histopathologic changes showed resolving tendencies over time in the single/acute exposure model, the inflammation and fibrosis were also dose-dependent in both the single/acute and repeated/chronic exposure models. These results support the clinical findings that prolonged exposure to high cumulative doses of HDs increases the risk of developing HDALI.

In most previous studies on lung pathophysiology in animals, even though PFT and histopathologic changes were examined, the precise correlations between PFT and histopathologic changes have rarely been properly studied. This study measured and matched the results of PFT parameters and histopathologic inflammation and fibrosis scores in each mouse to examine the correlation between PFT and histopathologic changes. As a result, resistance and compliance parameters and histopathologic scores showed significant correlations at 2 weeks in the single instillation model and at 10 weeks in the five-time instillation model, suggesting that histopathologic changes caused by HDs induce clinical symptoms reflected by abnormalities in PFT among HDALI victims.

This study was designed to investigate the acute and chronic effects, as well as the dose and time response of PHMG-p on the lungs using physiologic and histopathologic methods. Through this design, valuable results and insights were obtained, revealing important physiologic and histopathologic pathogenesis of HDALI. Therefore, the design and overall results of this study could be importantly referenced in future studies on toxic substances, particularly inhalational toxicity research.

There were some limitations in this experimental study. First, even though clinical HDALI is caused by inhalation of HDs, this study adopted IT instillation of PHMG-p. In studies using small rodents to model clinical respiratory diseases in humans, inhalational methods of delivering injurious substances cannot induce sufficient damage to the lungs. Due to the very narrow calibers of the upper and lower respiratory tracts compared to those of humans, most inhalational droplets cannot reach the terminal airways and lung parenchyma, even with equipment that produces small droplet sizes. Therefore, nasal or IT instillation methods are traditionally adopted for the study of inhalational toxic substances, and the results obtained using these methods have been acknowledged as appropriate substitutes for those from inhalational methods. Second, there were inconsistencies among PFT parameters, as well as within a single parameter depending on the dose and time. These are considered an unavoidable outcome due to the inability to conduct experiments with a sufficiently large number of animals.

In conclusion, the model investigating the acute and chronic effects, as well as the dose and time response of PHMG-p on the lungs in mice using PFT and histopathologic examinations, demonstrated that PHMG-p exposure in mice resulted in a restrictive ventilation defect with altered lung resistance and compliance, along with histopathologic lung inflammation and fibrosis.

## Figures and Tables

**Table 1:** The results of pulmonary function test parameters.

	Single PHMG-p Instillation Model							Five-Time PHMG-p Instillation Model		
		2 Weeks		4 Weeks		8 Weeks			10 Weeks	
Parameters	(Sub) Groups (PHMG-p, mg/kg)	Mean ± SD	*p*-Value	Mean ± SD	*p*-Value	Mean ± SD	*p*-Value	(Sub) Groups (PHMG-p, mg/kg)	Mean ± SD	*p*-Value
FEV100 (mL)	Saline	0.101 ± 0.003		0.100 ± 0.003		0.107 ± 0.003		Saline	0.105 ± 0.002	
	0.3	0.094 ± 0.002	0.004 *	0.104 ± 0.008	0.741	0.106 ± 0.003	0.962	0.1	0.096 ± 0.003	<0.001 *
	1	0.095 ± 0.002	0.012 *	0.099 ± 0.007	0.999	0.103 ± 0.002	0.087	0.3	0.098 ± 0.002	0.001 *
	3	0.098 ± 0.004	0.477	0.100 ± 0.008	0.999	0.102 ± 0.002	0.015 *	1	0.070 ± 0.006	
FEV100/FVC	Saline	0.936 ± 0.006		0.928 ± 0.007		0.948 ± 0.006		Saline	0.940 ± 0.007	
	0.3	0.925 ± 0.008	0.094	0.913 ± 0.018	0.215	0.948 ± 0.007	1.000	0.1	0.941 ± 0.010	0.983
	1	0.935 ± 0.007	0.994	0.907 ± 0.015	0.052	0.952 ± 0.007	0.865	0.3	0.936 ± 0.009	0.637
	3	0.933 ± 0.011	0.904	0.915 ± 0.008	0.334	0.950 ± 0.015	0.966	1	0.906 ± 0.037	
FVC (mL)	Saline	0.107 ± 0.004		0.109 ± 0.006		0.112 ± 0.004		Saline	0.111 ± 0.003	
	0.3	0.102 ± 0.003	0.127	0.114 ± 0.011	0.746	0.113 ± 0.003	0.982	0.1	0.102 ± 0.003	<0.001 *
	1	0.102 ± 0.003	0.173	0.110 ± 0.007	0.997	0.109 ± 0.003	0.332	0.3	0.106 ± 0.002	0.008 *
	3	0.105 ± 0.006	0.677	0.110 ± 0.009	0.999	0.107 ± 0.003	0.057	1	0.077 ± 0.004	
FRC (mL)	Saline	0.255 ± 0.019		0.222 ± 0.047		0.281 ± 0.052		Saline	0.191 ± 0.045	
	0.3	0.217 ± 0.028	0.320	0.214 ± 0.041	0.985	0.335 ± 0.019	0.062	0.1	0.178 ± 0.015	0.916
	1	0.254 ± 0.018	1.000	0.218 ± 0.028	0.998	0.292 ± 0.026	0.944	0.3	0.187 ± 0.042	0.839
	3	0.256 ± 0.065	1.000	0.213 ± 0.041	0.982	0.281 ± 0.030	1.000	1	0.156 ± 0.123	
TV (mL)	Saline	0.148 ± 0.003		0.171 ± 0.003		0.171 ± 0.004		Saline	0.165 ± 0.003	
	0.3	0.144 ± 0.001	0.030 *	0.168 ± 0.005	0.383	0.173 ± 0.004	0.816	0.1	0.167 ± 0.002	0.385
	1	0.143 ± 0.003	0.010 *	0.171 ± 0.003	0.998	0.168 ± 0.001	0.517	0.3	0.163 ± 0.005	0.759
	3	0.138 ± 0.003	<0.001 *	0.167 ± 0.003	0.284	0.167 ± 0.004	0.248	1	0.139 ± 0.003	
VC (mL)	Saline	0.831 ± 0.067		0.768 ± 0.057		0.778 ± 0.065		Saline	0.824 ± 0.089	
	0.3	0.724 ± 0.036	0.009 *	0.812 ± 0.089	0.750	0.876 ± 0.072	0.089	0.1	0.711 ± 0.045	0.042 *
	1	0.794 ± 0.041	0.613	0.821 ± 0.050	0.635	0.760 ± 0.078	0.965	0.3	0.752 ± 0.077	0.230
	3	0.760 ± 0.057	0.112	0.771 ± 0.097	1.000	0.759 ± 0.052	0.962	1	0.315 ± 0.036	
IC (mL)	Saline	0.757 ± 0.077		0.677 ± 0.040		0.685 ± 0.087		Saline	0.713 ± 0.065	
	0.3	0.640 ± 0.024	0.006 *	0.666 ± 0.049	0.992	0.789 ± 0.082	0.095	0.1	0.625 ± 0.051	0.071
	1	0.666 ± 0.031	0.037 *	0.698 ± 0.070	0.936	0.711 ± 0.050	0.928	0.3	0.605 ± 0.071	0.025 *
	3	0.610 ± 0.064	0.001 *	0.639 ± 0.083	0.736	0.663 ± 0.064	0.952	1	0.216 ± 0.025	
TLC (mL)	Saline	1.013 ± 0.088		0.900 ± 0.089		0.942 ± 0.108		Saline	0.898 ± 0.104	
	0.3	0.877 ± 0.073	0.019 *	0.846 ± 0.140	0.873	0.963 ± 0.107	0.973	0.1	0.770 ± 0.050	0.080
	1	0.953 ± 0.051	0.491	0.875 ± 0.089	0.984	0.947 ± 0.078	1.000	0.3	0.847 ± 0.115	0.627
	3	0.893 ± 0.072	0.043 *	0.860 ± 0.159	0.943	0.852 ± 0.025	0.303	1	0.357 ± 0.117	
Cchord	Saline	0.048 ± 0.003		0.042 ± 0.003		0.031 ± 0.006		Saline	0.034 ± 0.002	
(mL/cmH_2_O)	0.3	0.040 ± 0.002	0.001 *	0.044 ± 0.005	0.903	0.035 ± 0.004	0.503	0.1	0.029 ± 0.002	<0.001 *
	1	0.039 ± 0.003	<0.001 *	0.043 ± 0.006	0.937	0.030 ± 0.006	0.963	0.3	0.028 ± 0.001	<0.001 *
	3	0.037 ± 0.003	<0.001 *	0.041 ± 0.005	0.999	0.029 ± 0.002	0.833	1	0.011 ± 0.000	
Cfvc50	Saline	0.056 ± 0.005		0.053 ± 0.011		0.051 ± 0.007		Saline	0.050 ± 0.009	
(mL/cmH_2_O)	0.3	0.049 ± 0.005	0.132	0.048 ± 0.007	0.697	0.058 ± 0.007	0.503	0.1	0.046 ± 0.003	0.512
	1	0.051 ± 0.003	0.480	0.050 ± 0.008	0.841	0.048 ± 0.006	0.947	0.3	0.038 ± 0.007	0.024 *
	3	0.046 ± 0.007	0.020 *	0.049 ± 0.005	0.812	0.047 ± 0.009	0.912	1	0.013 ± 0.001	
Cdyn	Saline	0.027 ± 0.001		0.026 ± 0.002		0.027 ± 0.002		Saline	0.027 ± 0.001	
(mL/cmH_2_O)	0.3	0.023 ± 0.001	0.001 *	0.024 ± 0.004	0.392	0.028 ± 0.002	0.841	0.1	0.024 ± 0.001	0.007 *
	1	0.023 ± 0.002	0.008 *	0.027 ± 0.001	0.989	0.027 ± 0.003	0.999	0.3	0.025 ± 0.002	0.104
	3	0.018 ± 0.002	<0.001 *	0.023 ± 0.002	0.273	0.023 ± 0.006	0.417	1	0.009 ± 0.000	
RI	Saline	1.261 ± 0.064		1.239 ± 0.059		1.129 ± 0.084		Saline	1.142 ± 0.056	
(cmH_2_O/mL/s)	0.3	1.285 ± 0.045	0.940	1.320 ± 0.078	0.564	1.179 ± 0.033	0.749	0.1	1.244 ± 0.072	0.071
	1	1.240 ± 0.119	0.960	1.296 ± 0.169	0.794	1.161 ± 0.054	0.918	0.3	1.172 ± 0.090	0.755
	3	1.369 ± 0.032	0.078	1.388 ± 0.084	0.106	1.238 ± 0.138	0.164	1	2.134 ± 0.187	
dPmax	Saline	6.169 ± 0.198		7.396 ± 0.225		7.381 ± 0.638		Saline	6.883 ± 0.181	
(cmH_2_O)	0.3	6.997 ± 0.207	0.005 *	7.907 ± 1.605	0.774	6.705 ± 0.404	0.364	0.1	7.433 ± 0.364	0.039 *
	1	6.710 ± 0.454	0.087	6.981 ± 0.163	0.863	7.213 ± 0.274	0.975	0.3	7.290 ± 0.450	0.143
	3	8.109 ± 0.514	<0.001 *	8.024 ± 0.869	0.646	7.272 ± 1.148	0.993	1	15.791 ± 0.090	

One-way analysis of variance (ANOVA) test was performed for the statistical results. Data are represented as mean ± SD. *p*-values are for saline group versus PHMG-p subgroups. * *p* < 0.05. PHMG-p: polyhexamethylene guanidine hydrochloride-phosphate; FEV100: forced expiratory volume in 100 milliseconds; FEV100/FVC: forced expiratory volume in 100 milliseconds/forced vital capacity; FVC: forced vital capacity; FRC: functional residual capacity; TV: tidal volume; VC: vital capacity; IC: inspiratory capacity; TLC: total lung capacity; Cchord: chord compliance; Cfvc50: compliance at 50% vital capacity; Cdyn: dynamic compliance; RI: lung resistance; dPmax: the maximum pressure change over the breath.

**Table 2:** Histopathologic results in the single PHMG-p instillation model.

At 2 Weeks				
(Sub) Groups	Inflammation Score		Fibrosis Score	
	Mean ± SD	*p*-Value	Mean ± SD	*p*-Value
Saline	0.333 ± 0.816		0.167 ± 0.408	
PHMG-p 0.3 mg/kg	2.667 ± 0.516	<0.001 *	3.333 ± 1.211	<0.001 *
PHMG-p 1 mg/kg	3.000 ± 0.000	<0.001 *	4.500 ± 0.548	<0.001 *
PHMG-p 3 mg/kg	3.833 ± 0.408	<0.001 *	5.000 ± 0.632	<0.001 *
**At 4 Weeks**				
Saline	0.000 ± 0.000		0.00 ± 0.00	
PHMG-p 0.3 mg/kg	1.333 ± 1.033	0.006 *	1.333 ± 1.033	0.019 *
PHMG-p 1 mg/kg	2.833 ± 0.408	<0.001 *	3.667 ± 0.816	<0.001 *
PHMG-p 3 mg/kg	3.333 ± 0.516	<0.001 *	4.333 ± 0.516	<0.001 *
**At 8 Weeks**				
Saline	0.000 ± 0.000		0.000 ± 0.000	
PHMG-p 0.3 mg/kg	1.500 ± 1.225	0.007 *	1.000 ± 0.894	0.060 *
PHMG-p 1 mg/kg	2.500 ± 0.548	<0.001 *	2.833 ± 0.753	<0.001 *
PHMG-p 3 mg/kg	3.167 ± 0.408	<0.001 *	4.333 ± 0.516	<0.001 *

One-way analysis of variance (ANOVA) test was performed for the statistical results. Data are represented as mean ± SD. *p*-values are for saline group versus PHMG-p subgroups. * *p* < 0.05. PHMG-p: polyhexamethylene guanidine hydrochloride-phosphate.

**Table 3:** Histopathologic results at 10 weeks in the five-time PHMG-p instillation model.

(Sub) Groups	Inflammation Score		Fibrosis Score	
	Mean ± SD	*p*-Value	Mean ± SD	*p*-Value
Saline	0.333 ± 0.816		0.167 ± 0.408	
PHMG-p 0.1 mg/kg	3.000 ± 0.000	<0.001 *	3.167 ± 0.408	<0.001 *
PHMG-p 0.3 mg/kg	4.000 ± 0.894	<0.001 *	4.167 ± 0.753	<0.001 *
PHMG-p 1 mg/kg	6.000		6.000	

One-way analysis of variance (ANOVA) test was performed for the statistical results. Data are represented as mean ± SD. *p*-values are for saline group versus PHMG-p subgroups. * *p* < 0.05. PHMG-p: polyhexamethylene guanidine hydrochloride-phosphate.

**Table 4:** Correlation between PFT parameters and histopathologic scores.

At 2 Weeks in the Single PHMG-p Instillation Model										
	Cchord		Cfvc50		Cdyn		RI		dPmax	
	r	*p*-Value	r	*p*-Value	r	*p*-Value	r	*p*-Value	r	*p*-Value
Inflammation	−0.691	<0.001 *	−0.518	0.010 *	−0.701	<0.001 *	0.212	0.321	0.659	<0.001 *
Fibrosis	−0.771	<0.001 *	−0.556	0.005 *	−0.765	<0.001 *	0.348	0.095	0.737	<0.001 *
**At 10 Weeks in the Five-Time PHMG-p Instillation Model**										
Inflammation	−0.819	<0.001 *	−0.738	<0.001 *	−0.692	<0.001 *	0.567	0.009 *	0.593	0.006 *
Fibrosis	−0.787	<0.001 *	−0.687	<0.001 *	−0.671	0.001 *	0.577	0.008 *	0.588	0.006 *

One-way analysis of variance (ANOVA) test was performed for the statistical results. Data are represented as mean ± SD. *p*-values are for PFT parameters (Cchord, Cfvc50, Cdyn, RI, and dPmax) versus inflammation and fibrosis scores. * *p* < 0.05. PHMG-p: polyhexamethylene guanidine hydrochloride-phosphate; r: Pearson correlation coefficient; PFT: pulmonary function test; Cchord: chord compliance; Cfvc50: compliance at 50% vital capacity; Cdyn: dynamic compliance; RI: lung resistance; dPmax: the maximum pressure change over the breath.

## Data Availability

All data generated or analyzed during this study are included in this published article.
